# Pneumococcal meningitis secondary to chronic orbital inflammation with skull base erosion: a case report

**DOI:** 10.3389/fmed.2026.1918613

**Published:** 2026-09-30

**Authors:** Xinghua Tie, Xiaoman Sun

**Affiliations:** Department of Neurosurgery, Second Hospital & Clinical Medical School, Lanzhou University, Lanzhou, Gansu, China

**Keywords:** bacterial meningitis, chronic orbital inflammation, metagenomic next-generation sequencing, pneumococcal meningitis, skull-base erosion

## Abstract

This case report describes a 32-year-old female patient who developed acute pneumococcal meningitis following six-year chronic orbital inflammatory changes after strabismus corrective surgery. The patient experienced gradual deterioration of right-eye vision and recurrent retro-orbital headache; serial magnetic resonance imaging (MRI) from 2021 to 2023 demonstrated diffuse intraconal retrobulbar abnormalities and extraocular-muscle thickening. She subsequently presented with high fever, generalized seizures and impaired consciousness. Cerebrospinal fluid (CSF) metagenomic next-generation sequencing (mNGS) detected *Streptococcus pneumoniae* and low-abundance *Epstein–Barr virus* (EBV) DNA; Active EBV-associated central nervous system infection could not be confirmed in the absence of CSF quantitative EBV polymerase chain reaction (PCR), EBV serology and immune-status testing. The patient received broad-spectrum intravenous antimicrobial, antiviral and glucocorticoid therapy, followed by endoscopic endonasal resection of inflammatory lesions at the ethmoid-skull-base complex. Histopathological examination of surgical specimens revealed non-specific inflammatory granulation tissue, and Langerhans cell histiocytosis was excluded. At six-month follow-up, the patient remained seizure-free; infectious foci showed near-complete resolution on imaging, yet mild residual orbital-leptomeningeal abnormalities persisted with modest visual improvement. This case demonstrates the potential for delayed intracranial extension from long-standing chronic orbital inflammation and highlights both the diagnostic utility and interpretive limitations of CSF-mNGS in clinical practice.

## Case description

A 32-year-old woman was referred to the Department of Neurosurgery, Lanzhou University Second Hospital, with sudden severe headache and progressive decline in consciousness over six days. Six years earlier she had undergone strabismus corrective surgery. Postoperatively, right-eye best-corrected visual acuity deteriorated progressively from 0.8 to 0.3, accompanied by intermittent retro-orbital headaches. Serial brain MRI from 2021 to 2023 demonstrated diffuse abnormal T2-hyperintense signal in the right retrobulbar intraconal space, with thickening of the right medial, lateral, superior, and inferior rectus muscles and the superior oblique muscle, consistent with an inflammatory infiltrative process (Figure 1). She received multiple empirical courses of antimicrobials, which transiently relieved headaches but did not restore vision. Three years before admission she experienced a single generalized tonic–clonic seizure controlled with short-course antiepileptic drugs (AEDs); maintenance therapy was not continued. During this six-year interval, no formal ophthalmological quantification of visual acuity at each visit, ocular motility testing, proptosis measurement, pupillary examination, fundoscopy, colour vision, or visual field testing was documented. Likewise, serological evaluation to exclude thyroid orbitopathy, immunoglobulin G4 (IgG4)-related disease, idiopathic orbital inflammation, granulomatous disorders, lymphoma, sarcoidosis, vasculitis, or chronic infection was not reported.

**Figure 1:**
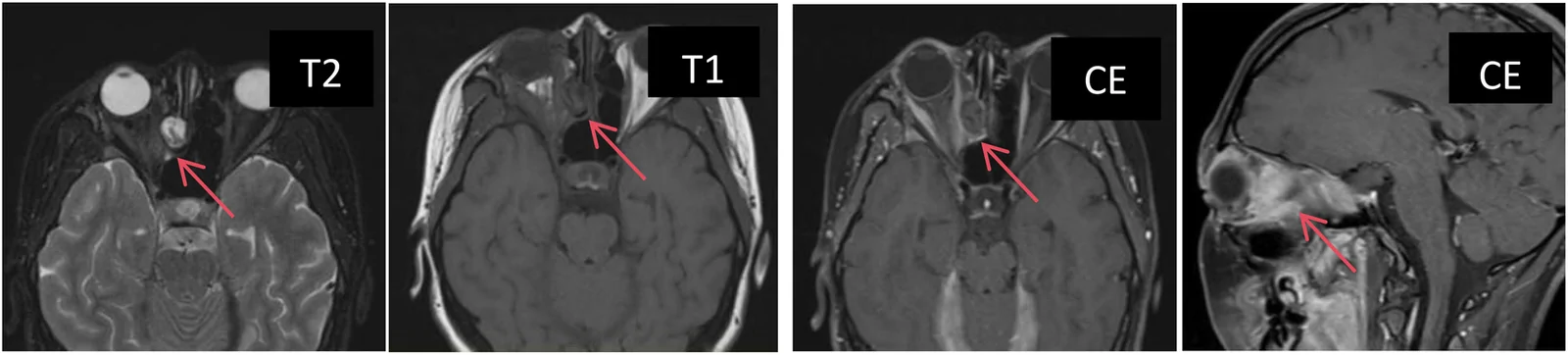
Axial and coronal orbital MRI (2023, T2-weighted sequences). Diffuse T2-hyperintense signal occupies the right intraconal compartment with circumferential thickening of multiple extraocular muscles. Arrows mark thinning of medial orbital wall secondary to chronic inflammatory infiltration, without discrete intra-orbital abscess.

Ten days before admission she developed cough and low-grade fever, for which she took undocumented oral antimicrobial medications at home. Six days later, acute headache and impaired consciousness prompted presentation to a local hospital. Cranial computed tomography (CT) showed hyperdensity along the falx cerebri and tentorium cerebelli, initially misinterpreted as subarachnoid haemorrhage (SAH). Digital subtraction angiography (DSA) raised the possibility of a left C7 aneurysm. Following a generalized seizure with worsening consciousness she was intubated and received intracranial pressure (ICP) reduction, sedation, analgesia, dual AEDs (levetiracetam 0.5 g every 12 h and oxcarbazepine 0.3 g every 12 h). Persistent high fever (40 °C) prompted lumbar puncture before any intravenous antibiotics were administered at that treating institution, yielding bloody CSF. Initial CSF analysis showed white blood cell count (WBC) 808 × 10^6^/L [mononuclear cells (MN) 19 × 10^6^/L, polymorphonuclear neutrophils (PMN) 81 × 10^6^/L], total protein 1.503 g/L, glucose 6.95 mmol/L, with simultaneous serum glucose 10.2 mmol/L (CSF-to-serum ratio 0.68). The opening pressure and red blood cell count were not recorded. CSF Gram stain revealed no microorganisms, and 14-day conventional bacterial culture remained negative, most likely attributable to undocumented pre-hospital oral antimicrobial intake. A CSF sample was sent for mNGS. During inter-hospital transfer she had a further generalized seizure with fever peaking at 41 °C.

On admission, vital signs: temperature 39.5 °C, heart rate 86 bpm, respiratory rate 28 bpm, blood pressure 107/63 mmHg. Glasgow Coma Scale score E1VTM4 with marked nuchal rigidity. Bilateral pupil diameter was 2.5 mm; the right pupillary light reflex was absent and the left sluggish. Limb muscle tone was normal, but formal muscle-strength testing was not feasible due to poor cooperation. Peripheral-blood investigations showed leukocytosis (WBC 13.09 × 10^9^/L, neutrophils 9.72 × 10^9^/L) and elevated procalcitonin (0.823 ng/mL). Blood cultures were negative. CT confirmed cerebral oedema with sulcal effacement and again demonstrated hyperdensity along the falx and tentorium, which was considered more compatible with pseudo-subarachnoid haemorrhage in the setting of severe meningitis and cerebral oedema, accompanied by skull-base erosion (Figure 2). CT angiography (CTA) excluded aneurysm. Electroencephalography (EEG) demonstrated an alternating background pattern: polymorphic delta activity (state 1) and theta activity (state 2), with absent reactivity (Figure 3).

**Figure 2:**
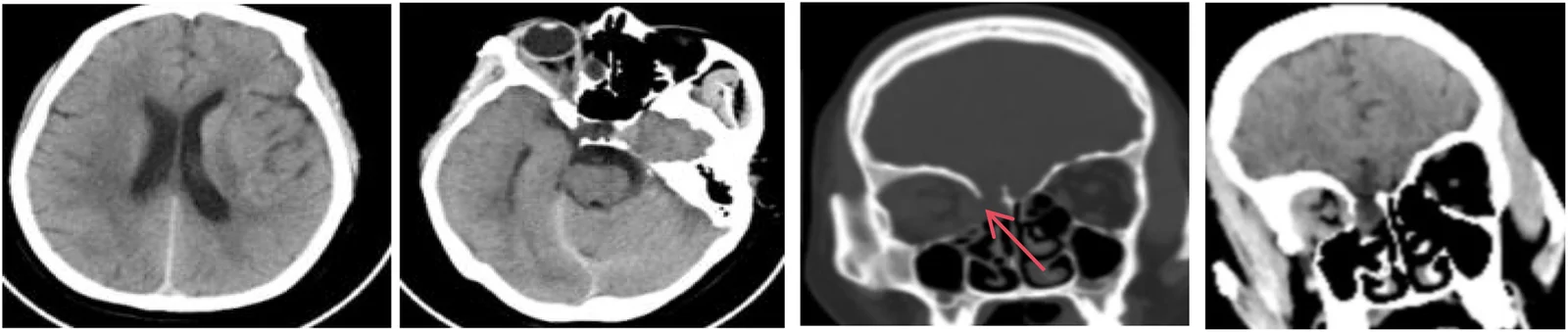
Brain CT (brain-window and supplementary bone-window reconstruction, admission day). Sulcal effacement over bilateral frontal, parietal, and occipital lobes indicates cerebral oedema. Hyperdensity is seen along the falx cerebri and tentorium cerebelli. Arrows on bone-window panels demonstrate skull-base bony erosion.

**Figure 3:**
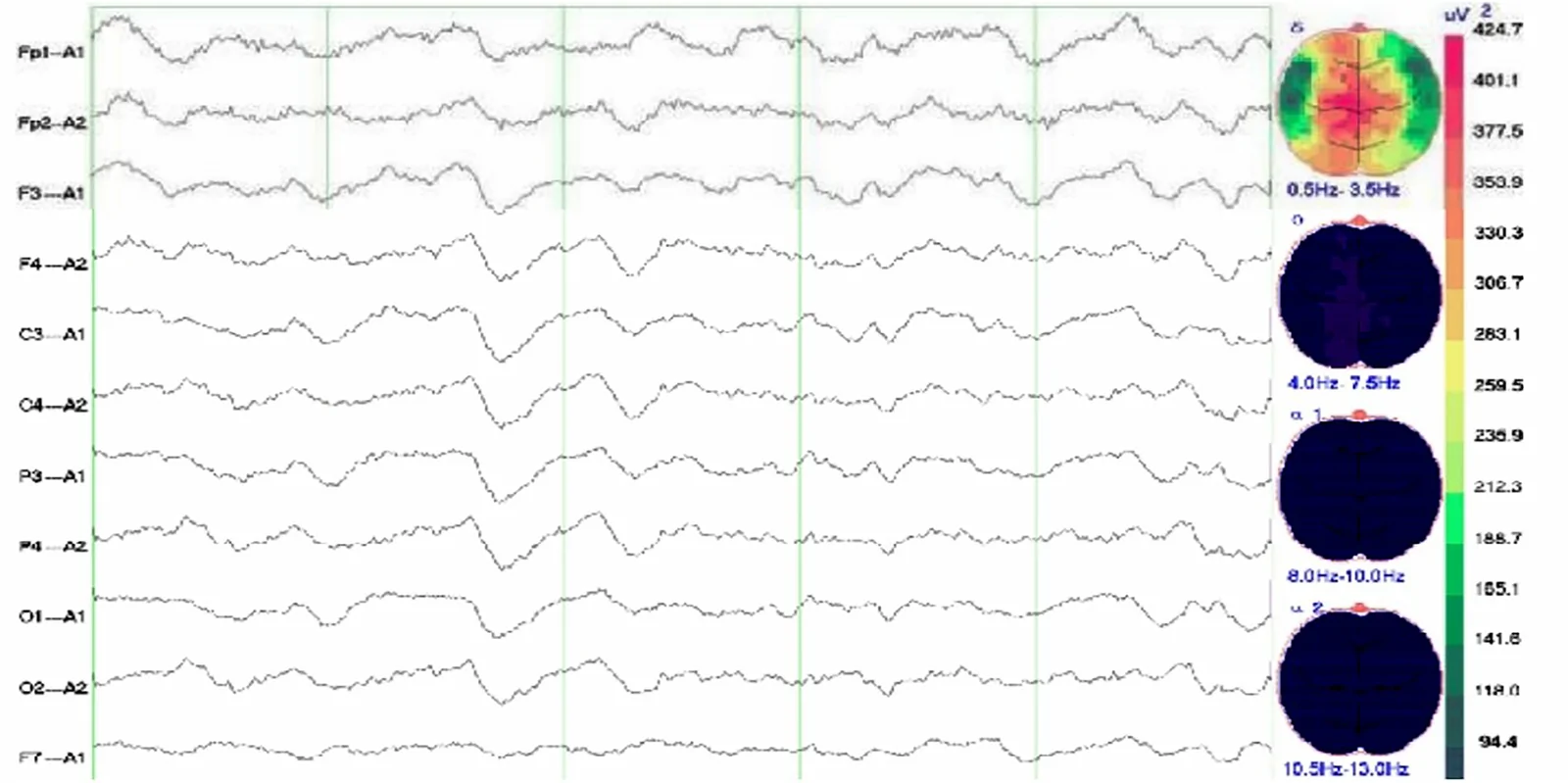
EEG (admission day, referential montage) demonstrating background activity in an alternating pattern. State 1: polymorphic delta wave activity dominates all leads. State 2: theta wave activity dominates all leads. EEG reactivity is absent.

CSF mNGS results from the outside hospital identified *S. pneumoniae* (3,261 reads) and EBV (368 reads). The patient weighed 58 kg, with baseline serum creatinine of 62 μmol/L (eGFR > 90 mL/min/1.73 m^2^). Broad-spectrum intravenous therapy was commenced: vancomycin 1 g every 8 h plus meropenem 2 g every 8 h for 14 days, acyclovir 0.5 g every 8 h for 10 days, and dexamethasone 5 mg every 12 h for 7 days. After four doses of vancomycin, the trough serum vancomycin concentration was 16 µg/mL and the trough CSF concentration was 7 µg/mL; daily renal function and urine output monitoring remained normal, and therefore no dose adjustment was required. The rationale for choosing mNGS over a targeted multiplex bacterial and viral meningitis PCR panel was not documented; a multiplex meningitis PCR assay was unavailable at our institution at the time. Over the subsequent week her consciousness gradually cleared, fever resolved, and peripheral leukocytosis normalized.

Repeat lumbar puncture on hospital day 13 showed an opening pressure of 150 mmH_2_O, clear CSF, glucose 8.25 mmol/L with simultaneous serum glucose 9.4 mmol/L (ratio 0.88), total protein 0.45 g/L, WBC 13.0 × 10^6^/L, and no red blood cells. Culture was again negative. Antibiotics were de-escalated to intravenous ceftriaxone 2 g every 12 h for a further two weeks. The patient was extubated on day 5 and transferred out of the intensive care unit after ten consecutive afebrile days.

Brain MRI revealed a right ethmoid sinus mass invading the anterior skull base and extending into the right orbit (Figure 4). On hospital day 17 she underwent endoscopic endonasal resection of the skull-base lesion, primarily for infectious source control, diagnostic confirmation, and skull-base reconstruction to prevent CSF leakage and recurrent intracranial contamination. Intraoperatively, full-thickness ethmoid bone destruction was observed, with necrotic and inflammatory granulation tissue herniating through defects in the anterior ethmoid and medial orbital wall. All abnormal tissue was resected, and the skull base was reconstructed with artificial dura and a pedicled middle turbinate mucosal flap; no intraoperative CSF leak occurred. Postoperative CT showed no significant haemorrhage (Figure 5).

**Figure 4:**
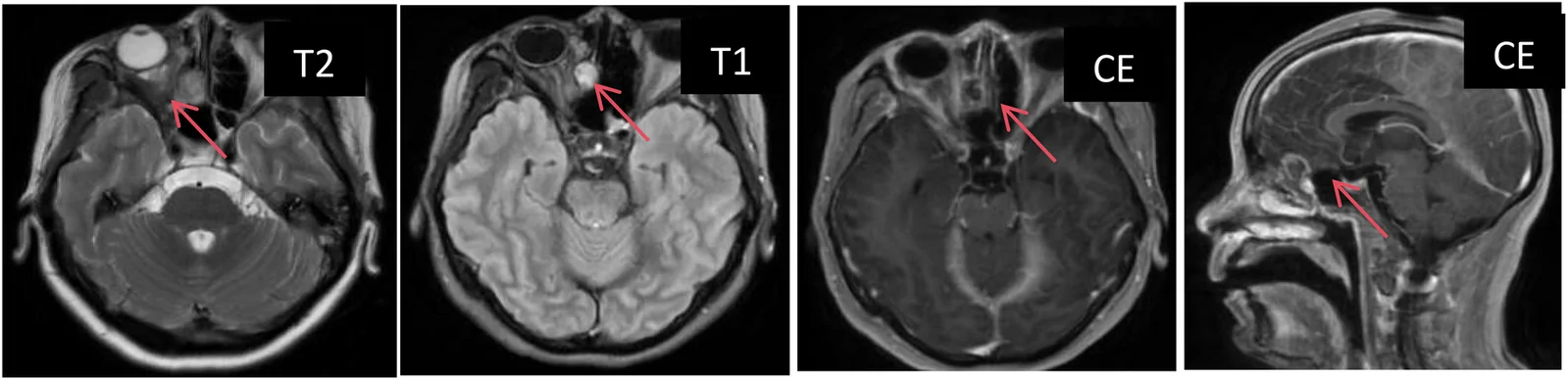
Brain MRI (hospital day 10; axial and coronal planes; T2, T1, and post-contrast T1-weighted sequences). A right ethmoid sinus mass with local invasion of the anterior skull base and extension into the right orbit is shown. Arrows indicate the enhancing lesion and skull-base defect.

**Figure 5:**
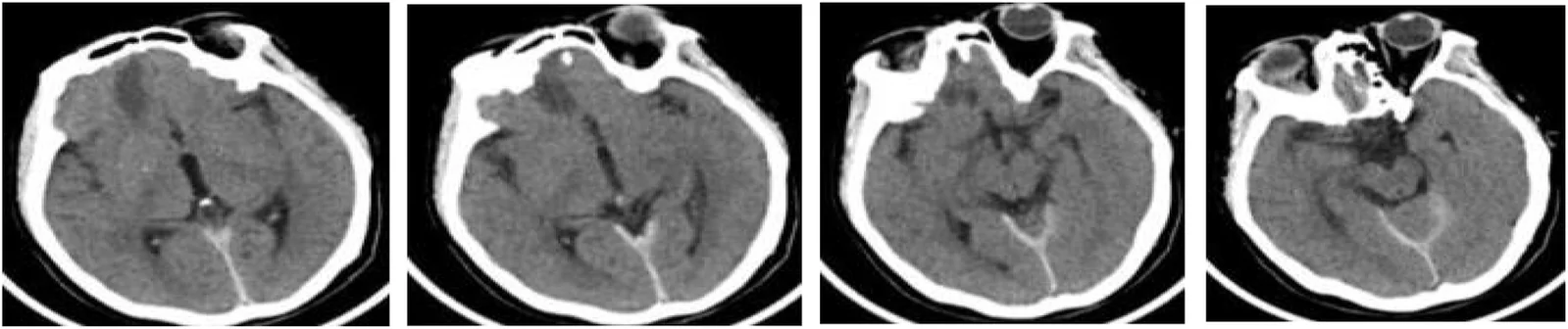
Postoperative CT (hospital day 18, brain-window and bone-window reconstruction) following skull-base lesion resection. A new low-density area is seen in the right frontal region (arrows mark). Thickening of the right medial rectus, lateral rectus, and optic nerve persists compared with prior imaging. Increased density along the falx cerebri and tentorium cerebelli appears similar to the previous scan.

Histopathological examination of the resected ethmoid–skull base specimen demonstrated liquefied brain tissue, proliferating small blood vessels, granulation tissue, ciliated columnar epithelium, and extensive infiltration by inflammatory cells and histiocytes. Immunohistochemistry was positive for cytokeratin (ciliated columnar epithelium), leukocyte common antigen (lymphocytes), CD68 and CD163 (histiocytes), synaptophysin (brain tissue), S-100 and glial fibrillary acidic protein (glia), CD34 and CD31 (vasculature), and Neu-N (neurons), with a Ki-67 proliferation index of 2%. No microbial colonies or viral inclusions were seen on special stains, and tissue culture was not performed. Langerhans cell histiocytosis was formally considered but excluded based on combined histomorphology and clinical history, confirming that skull-base bone erosion was inflammatory rather than neoplastic (Figure 6).

**Figure 6:**
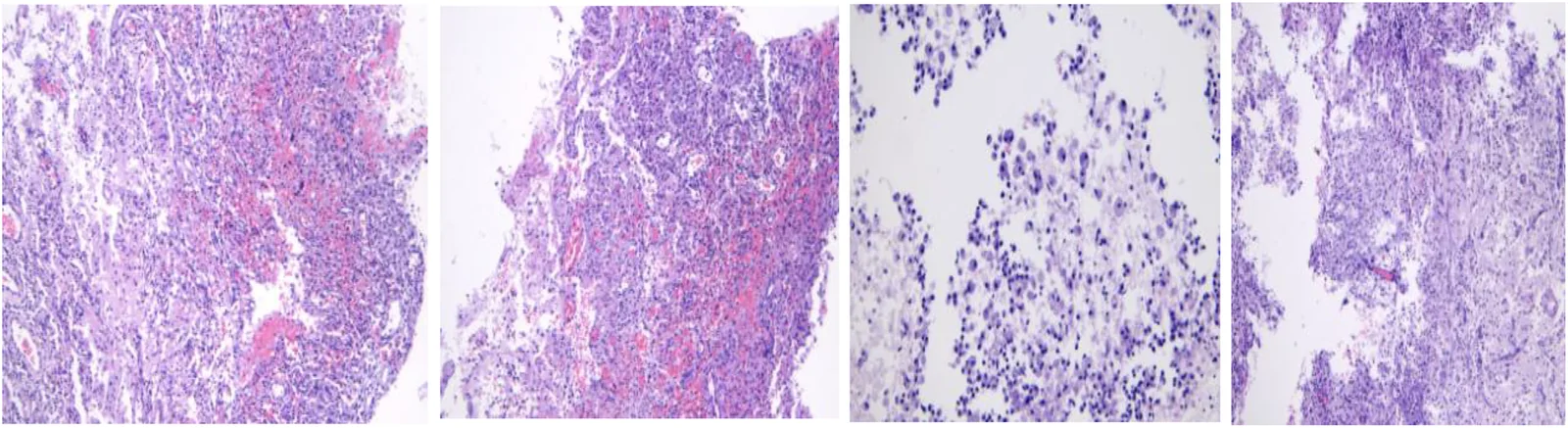
Histopathology of the resected ethmoid–skull base specimen (hospital day 17) showing liquefied brain tissue, proliferating blood vessels, granulation tissue, and ciliated columnar epithelium, with extensive infiltration by inflammatory cells and histiocytes. Immunohistochemistry: CKp (ciliated columnar epithelium +), LCA (lymphocytes +), CD68 (histiocytes +), CD163 (histiocytes +), Syn (brain tissue +), S-100 (glia +), GFAP (glia +), CD34 (vasculature +), CD31 (+), Neu-N (neurons +), Ki-67 (2%+).

By postoperative day 28 the patient was fully conscious, afebrile, with normal laboratory parameters, and had experienced no further seizures. She was discharged on oral levetiracetam 0.5 g twice daily, tapered over three months and discontinued without seizure recurrence. At six-month follow-up, retro-orbital headache had resolved and right-eye visual acuity had improved modestly to 0.4. MRI demonstrated near-complete resolution of the dominant intracranial infectious focus, yet a small ring-enhancing nodule persisted within the right ethmoid surgical cavity, along with residual right optic nerve sheath effusion and mild extraocular muscle thickening. Linear leptomeningeal and dural enhancement also persisted (Figure 7). She had returned to full-time work without fever or seizures.

**Figure 7:**
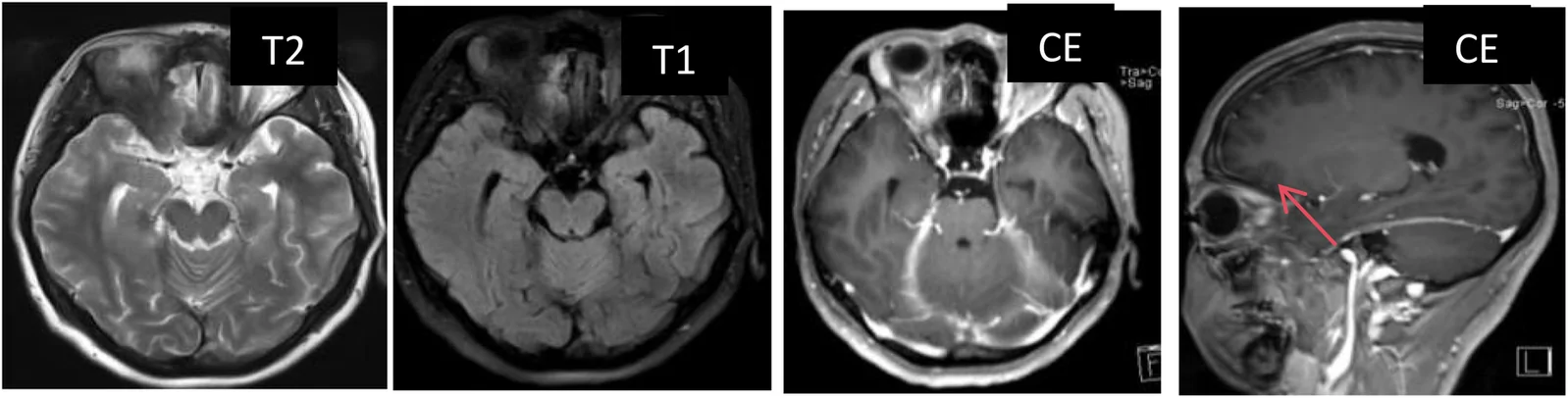
Follow-up MRI (six months postoperatively) after right ethmoid sinus–anterior skull base lesion resection. A ring-enhancing nodule persists within the right ethmoid surgical cavity. Right optic nerve sheath effusion and thickening of the right medial rectus muscle are present. Linear T2-shortening and band-like enhancement along the interhemispheric fissure and supratentorial region; leptomeningeal thickening and enhancement in the right parietal and bilateral frontal sulci, similar to prior. Slight thickening and enhancement of the dura over bilateral fronto-temporo-parietal regions, similar to prior. Previously noted punctate parenchymal enhancements have essentially resolved (arrows).

## Discussion

This case illustrates a rare trajectory from chronic orbital inflammation to pneumococcal meningitis via contiguous skull-base erosion. The six-year interval between strabismus surgery and acute intracranial infection raises the possibility of an occult postoperative nidus; however, causality cannot be established without operative records detailing the surgical approach, implanted materials, and anatomical correlation. The prior surgery is therefore presented only as a plausible association rather than a demonstrated risk factor.

The chronic orbital process was diagnosed radiologically as retrobulbar inflammation with extraocular muscle thickening. The term “chronic retrobulbar myositis” implies a specific histopathological diagnosis that was never confirmed by biopsy. The differential diagnosis is broad, encompassing thyroid orbitopathy, IgG4-related orbital disease, idiopathic orbital inflammation, granulomatosis with polyangiitis, sarcoidosis, lymphoma, and chronic infection. Our patient lacked formal ophthalmological characterization and autoimmune or infectious serological screening during the chronic phase, representing a substantial diagnostic gap. The final surgical pathology—nonspecific granulation tissue with inflammation, vascular proliferation, and entrapped ciliated epithelium—did not confirm myositis or identify a causative organism. This entity is therefore best described as chronic orbital inflammatory disease of uncertain aetiology.

The diagnosis of pneumococcal meningitis was supported by acute fever, nuchal rigidity, impaired consciousness, neutrophil-predominant CSF pleocytosis, and high-abundance *S. pneumoniae* detection by mNGS (1–3). Several microbiological observations require discussion. Initial and follow-up CSF-to-serum glucose ratios were 0.68 and 0.88 respectively. Classically, bacterial meningitis presents with a CSF-to-serum glucose ratio below 0.4. Preserved ratios in this case may be explained by partial bacterial suppression from pre-hospital oral antimicrobials, as well as increased blood–CSF-barrier permeability caused by severe meningeal inflammation. A non-decreased CSF-to-serum glucose ratio therefore cannot rule out pneumococcal meningitis when supported by clinical manifestations, neutrophilic CSF pleocytosis and high-confidence mNGS pathogen detection. Conventional bacterial cultures were negative, most likely due to undocumented pre-hospital oral antimicrobial exposure. The bloody appearance of the initial CSF raises three possibilities—traumatic tap, true subarachnoid haemorrhage, or inflammatory vascular leakage across eroded bone—that could not be rigorously distinguished retrospectively.

The clinical significance of 368 EBV reads is uncertain. EBV DNA in CSF may represent active CNS infection, systemic reactivation, or incidental shedding. Definitive diagnosis of EBV meningoencephalitis requires quantitative EBV PCR in CSF, plasma EBV DNA load, EBV serology (viral capsid antigen IgM/IgG, Epstein–Barr nuclear antigen IgG), and assessment of immune status including HIV testing. None was performed (4, 5). Moreover, acyclovir was administered concurrently with antibacterial therapy, dexamethasone, AEDs, and supportive care, precluding attribution of neurological improvement to any single intervention. This case is characterized as pneumococcal meningitis with concomitant CSF EBV detection, rather than mixed bacterial–viral meningoencephalitis (6).

CSF mNGS provides broad, unbiased pathogen detection and proved pivotal when conventional cultures were negative, yet it lacks standardized quantitative thresholds, cannot reliably distinguish active infection from colonisation or latent viral shedding, and yields no antimicrobial susceptibility data. A multiplex meningitis PCR panel might have confirmed *S. pneumoniae* faster and more cheaply while avoiding ambiguity around low-level EBV detection; however, this assay was unavailable at our institution, so CSF mNGS was used as the primary molecular diagnostic test. The choice was therefore driven by assay availability rather than a documented clinical rationale.

Endoscopic surgical resection served three complementary purposes: diagnostic histopathological confirmation, eradication of the infectious nidus, and skull-base reconstruction to seal the orbitocranial communication. Successful withdrawal of AEDs after infection control supports the interpretation that seizures were symptomatic of acute meningeal inflammation and structural irritation rather than reflecting an underlying epileptogenic focus.

## Limitations

This report has several acknowledged limitations. Preoperative orbital biopsy was not performed, precluding histopathological confirmation of myositis and limiting the chronic orbital diagnosis to imaging-based inference. Confirmatory quantitative EBV PCR, EBV serology, and HIV testing were unavailable. No microbial culture or molecular pathogen testing was performed on the surgically resected specimen. The primary anatomical origin of the destructive process remains uncertain. A causal relationship between strabismus surgery and subsequent chronic orbital inflammation cannot be established without detailed operative records. Follow-up is limited to six months. Despite written informed consent for publication of clinical and imaging data, formal patient-reported perspective was not obtained because the patient declined structured interview for this case report.

## Conclusions

This case documents pneumococcal meningitis secondary to chronic orbital inflammation with skull-base erosion. CSF mNGS facilitated pathogen detection where conventional methods failed, yet its limitations—particularly the uncertain significance of low-level viral reads—must be recognised. Early targeted antimicrobial therapy combined with endoscopic surgical eradication of the infectious focus achieved a favourable neurological outcome. Clinicians should maintain a high index of suspicion for occult skull-base erosion in patients with chronic orbital inflammation and new neurological deficits.

## Data Availability

All data generated or analyzed during this case report are included in this published article.The underlying patient data (detailed medical history,examination findings, and laboratory reports) are not publicly available to protect patient privacy, in compliance with the ethical consent obtained. De-identified data may be shared upon reasonable request to the corresponding author, subject to approval by the Lanzhou University Second Hospital Ethics Committee. The original imaging studies and pathological specimens are retained in the departments of Radiology and Pathology at Lanzhou University Second Hospital and may be made available for review under the same conditions.
