# Supplementary material for: Pneumococcal meningitis secondary to chronic orbital inflammation with skull base erosion: a case report
Source: Front Med (Lausanne). 2026 Sep 30;13:1918613. doi: 10.3389/fmed.2026.1918613 (PMC13661423; doi:10.3389/fmed.2026.1918613)
Supplement: Supplementary file 1 [file Datasheet1.pdf]

Supplementary Material

Supplementary Table S1. Clinical Event Timeline

**Time point**	**Key clinical event**
6 years before admission	Strabismus corrective surgery; gradual right-eye visual loss and intermittent retro-orbital headache onset
2021–2023	Serial orbital MRI showing intraconal inflammatory changes and extraocular-muscle thickening
3 years before admission	Single generalized tonic–clonic seizure; short-course AEDs given without maintenance therapy
10 days before admission	Prodromal cough and low-grade fever; undocumented oral antimicrobials taken at home
6 days before admission	Acute headache, altered mental status; admission to local hospital; initial CT, DSA; lumbar puncture before local intravenous antibiotics; CSF mNGS testing
Hospital day 1 (our centre)	Transfer to neurosurgical department; ICU admission; initiation of broad-spectrum antimicrobial, antiviral and glucocorticoid therapy
Hospital day 5	Extubation
Hospital day 10	Brain MRI revealing ethmoid sinus mass invading anterior skull base
Hospital day 13	Second lumbar puncture; antibiotic de-escalation to ceftriaxone
Hospital day 17	Endoscopic endonasal resection of skull-base inflammatory lesion and skull-base reconstruction
Hospital day 18	Postoperative CT showing no significant haemorrhage
Postoperative day 28	Hospital discharge with oral levetiracetam
3 months post-discharge	Levetiracetam tapered and discontinued; no seizure recurrence
6-month follow-up	Seizure-free; retro-orbital headache resolved; right-eye visual acuity improved to 0.4; follow-up brain-orbital MRI
